# Acute adverse events from over-the-counter Chinese herbal medicines: a population-based survey of Hong Kong Chinese

**DOI:** 10.1186/1472-6882-13-336

**Published:** 2013-11-27

**Authors:** Jean H Kim, Elizabeth MS Kwong, Vincent CH Chung, John CO Lee, Terry Wong, William B Goggins

**Affiliations:** 1 School of Public Health and Primary Care, The Chinese University of Hong Kong, Prince of Wales Hospital, Shatin, New Territories, Hong Kong

**Keywords:** Traditional Chinese Medicine, Health Services, Health education, Prevention, Adverse events, Pharmaceutical safety, Complementary and alternative medicine, Traditional medicine

## Abstract

**Background:**

Although over-the-counter traditional Chinese herbal medicine (COTC) is commonly used to treat everyday illness in many parts of the world, no population-based study has been done to examine the prevalence and factors associated with COTC-related adverse events.

**Methods:**

A cross-sectional telephone survey was conducted among Hong Kong Chinese adults in 2011 (n = 1100) with informed verbal consent. Stepwise logistic regression of demographic, attitudinal and behavioral variables was used to determine factors associated with past-year adverse events.

**Results:**

Of study respondents, 71.7% (789/1100) reported past-year COTC use and 2.3% (25/1100) reported at least one COTC-related adverse event in the past year. Of the 27 adverse events cases reported among COTC users, the most common were allergic reactions (n = 11) dizziness (n = 5), and gastro-intestinal problems (n = 4). Pills/capsules were the dosage form that caused the highest proportion of adverse events (n = 10), followed by plasters (n = 7), creams/ointments (n = 5), and ingestible powders (n = 2).

Although COTC users reporting adverse events were more likely to report greater practices to avoid adverse events (OR = 6.47; 95% CI: 1.38-30.3); they were also more likely to possess lower education levels (OR = 9.64, 95% CI: 2.20-42.3) and to have received COTC information from non-reliable, mass-media information sources such as magazines (OR = 3.32; 95% CI: 1.01-8.50) or television (OR = 2.93; 95% CI: 1.03-10.7). Package labels were also felt to be unclear by 42.9% of COTC users. A large proportion of COTC users demonstrated low levels of COTC-related knowledge, while the main impediment to greater information-seeking was the belief that reliable COTC information is not obtainable from Western health professionals.

**Conclusions:**

Despite global movements toward more stringent complementary medicine regulation, the limited accessibility of reliable information and widespread misperceptions among consumers present major challenges for the safe use of complementary medicine.

## Background

The use of complementary and alternative medicine has been increasing worldwide in recent decades. One reason for its continued popularity is the pervasive belief that it is a safer alternative to conventional allopathic medicine [1]. In response to increasing popularity of complementary medicine, the World Health Organization has outlined a framework of action for greater integration of traditional medicine/complementary and alternative medicine into national health care systems [2]. Components of this World Health Organization Traditional Medicine Strategy included the regulation of herbal medicines and the promotion of rational traditional medicine use by consumers. In East Asian cultures, Traditional Chinese Medicine (TCM), as a form of complementary medicine, has long been commonplace for treating a spectrum of illnesses [3]. In recent years, TCM products have become more readily available in non-Asian countries [4-9]. By 2002, TCM exports from China were reported to be as high as 100 million USD per year [10,11].

Although TCM has a long tradition of use in Hong Kong, its regulation commenced only after the 1997 handover [12,13]. Hong Kong’s regulatory bodies have since classified Chinese herbal medicines into two general types of products [14]. The first type are processed medicinal materials for decoctions (Yin Pian) that are sold by licensed retailers [15]. These products are used for customized formulations based upon prescriptions from TCM doctors. The second type of TCM products are proprietary over-the-counter Chinese medicines (COTC) that do not require management by licensed purveyors. Similar to Western over-the-counter drugs, COTC are mass-manufactured and sold in finished dose form (such as pre-packaged pills or ointments).

Formal registration of TCM phytotherapeutic products in Hong Kong commenced in 1999. The Chinese Medicine Ordinance of 2003 mandated that all COTC products submit laboratory certification for formal registration and that all labels include information on dosage, active ingredients, indications, and contra-indications for use [16]. All COTC products must undergo full formal registration or be under a transitional licensure scheme in order to be sold legally in Hong Kong [17]. Of the 10,518 COTC products available in Hong Kong as of January 2012, only 188 (1.8%) have completed full formal registration [18], while the remaining products remain under the transitional scheme.

It is worthwhile to document adverse events in a population that is in the process of implementing formal COTC registration, in order to assess the effectiveness of these regulations and to document patterns of adverse events. Despite efforts to regulate TCM products in Hong Kong, reports of TCM poisoning cases have increased [19,20]. Studies conducted in the US and Asia have noted that many TCM drugs were compromised by adulteration with western pharmaceuticals such as sildenafil, with heavy metal contamination, with inadequate labeling, and with commercial counterfeiting [19-28]. In Hong Kong, as in other countries in the East Asia region, self-medication using over-the-counter TCM without consultation of TCM professionals is a widespread cultural practice for conditions ranging from the common cold to chronic health conditions [29,30]. Studies conducted in the past decade have reported that about one in seven TCM-related toxicity cases in Hong Kong were attributed to COTC use [19,20]. Past studies in Hong Kong, however, have been conducted on small non-representative samples [31] or from emergency room admissions [19,20,32]. These studies may present a highly biased picture of the COTC-related harms in the general population, since only severe adverse events are likely to be treated in emergency rooms. Internationally, studies on complementary medicine have noted relatively low levels of adverse drug reactions as compared to conventional Western drugs [33-35]. However, these studies of adverse events were typically conducted on patients who were prescribed complementary and alternative medicine by licensed practitioners and not self-medicating. Studies conducted in numerous countries have shown that self-medication, a noted risk factor for adverse events, is a common practice for allopathic as well as complementary medicine [36-40] but data on the prevalence of adverse events among self-medicating users of complementary medicine is limited [41].

In order to address the above knowledge gaps, this study examines the occurrence of COTC-related adverse events and factors that may predispose individuals for COTC adverse events in a population-based sample of Chinese adults. In Hong Kong, the difference between over-the-counter COTC and herbal TCM is well-known, since herbal TCM requires a prescription by a TCM doctor and customized preparation of the medicine, whereas COTC is sold in mass-manufactured, pre-dosed forms that do not require prescriptions. The study results can inform consumer regional COTC drug safety guidelines and offer insights for other governments that are in the process of strengthening COTC regulation.

## Methods

### The study population

**Figure 1:**
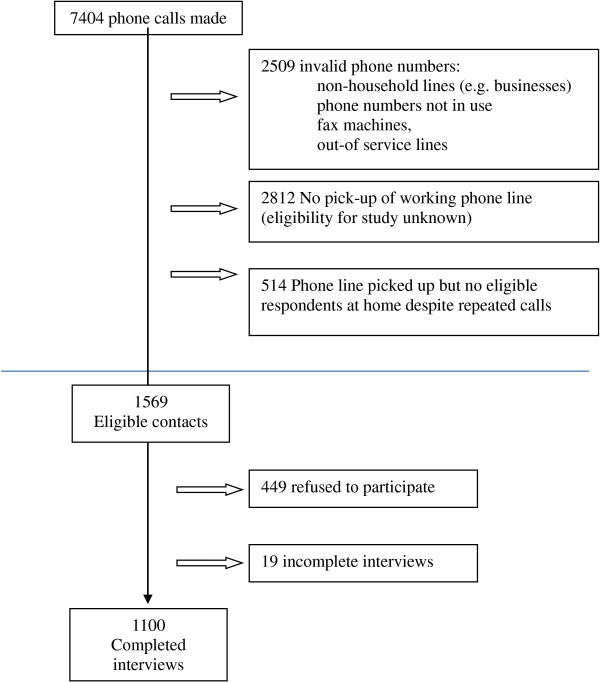
Study recruitment.

The target population was comprised of all Cantonese-speaking Hong Kong residents over the age of 18. After the preliminary survey instrument was anonymously pilot-tested on 50 respondents and revised for clarity and accuracy, an anonymous, random telephone survey was conducted in September 2011 using the ‘last birthday’ method of selecting respondents. For unanswered calls, at least 4 other independent calls were made. Trained interviewers interviewed 1100 respondents using colloquial Cantonese. The sample size was derived from power calculations to allow for adequate sample size to conduct multivariable logistic regression with ten covariates. After briefing the individual about the purpose of the survey, interviewers obtained verbal consent to participate in the study before conducting the interview. Research ethics approval was obtained from the ethics board of the sponsoring university. A flow chart of the study recruitment process is shown below (Figure 1). Of households with an eligible member, the overall response rate was 70.1%.

### Measurements

#### ***Socio-demographic and background information***

The socio-demographic and background information of respondents were recorded (see Table 1). Respondents were then asked whether they had used COTC products in the preceding 12 months. Respondents were asked about their knowledge about COTC safety, potential harms, side effects, and possible drug interactions between COTC with TCM or conventional Western drugs (see Table 1). Correct responses to these six items were summed to derive a summative knowledge score (range 0–6).

**Table 1:** Characteristics of the study respondents

	**COTC users**	**Non-users**	**P (χ**^**2**^**)**	**Total sample**	**Hong Kong population**
	**(n = 789) %**	**(n = 311) %**		**(n = 1100) % (95% CI)**	
Gender			0.284		
Male	46.3%	49.8%		47.3% (44.3-50.2)	46.6%^a^
Female	53.7%	50.2%		52.7% (49.9-55.7)	53.4%
Age group			0.782		
18 – 24 y	10.0%	9.0%		9.7% (8.0-11.5)	10.2%^a^
25 – 34 y	18.4%	21.5%		19.3% (16.9-21.6)	18.2%
35 – 44 y	19.5%	18.0%		19.1% (16.8-21.4)	19.0%
45 – 54 y	22.1%	20.3%		21.5% (19.1-24.0)	21.6%
55 – 64 y	15.3%	14.8%		15.2% (13.1-17.3)	15.4%
65 y or above	14.7%	16.4%		15.2% (13.9-17.3)	15.6%
Education			0.627		
No formal schooling/Primary	15.9%	16.8%		16.2% (13.9-18.3)	25.4%^b^
Secondary 1 - 7	55.5%	57.6%		56.1% (52.8-58.7)	51.5%
At least some post-secondary	28.6%	25.7%		27.6% (24.9-30.2)	22.9%
Household income (1 USD = 7.8 HKD)			< 0.001		
< 15000 HKD/month	22.5%	31.6%		25.1% (22.3-27.8)	43.1%^b^
15000 – 29999 HKD/month	46.2%	33.8%		42.8% (39.7-45.9)	30.0%
> 30000 HKD/month	31.2%	34.6%		32.2% (29.2-35.1)	26.9%
Employment status			0.402		
Employed at least part-time	51.2%	50.0%		50.9% (47.9-53.8)	52.1%^c^
Homemaker	25.0%	22.6%		24.3% (21.8-26.8)	18.5%
Other (student, unemployed, retired)	23.8%	27.4%		24.9% (29.2-35.1)	29.0%
Health insurance					
Have health insurance	52.3%	48.5%	0.263	51.2% (48.2-54.1)	NA
Self-perceived health status			<0.001		
Very good/Good	49.1%	55.2%		50.8% (47.8-53.7)	NA
About average	46.8%	38.5%		44.5% (41.5-47.4)	
Bad/Very bad	4.0%	6.5%		4.8% (3.5-6.0)	
COTC-related adverse event history					
Reported past year adverse event	3.2%	NA		2.3% (1.3-3.1)	NA
Correct response to COTC knowledge items (correct answer in parentheses)					
Cannot cause long term bodily damage (No)	24.0%	19.0%	0.09	22.7% (20.2-25.2)	NA
Can negatively interact w/western drugs (Yes)	80.5%	77.5%	0.224	79.5% (77.1-81.9)	NA
Can have interactions with herbal TCM (Yes)	51.6%	41.8%	0.003	48.9% (45.9-51.8)	NA
COTC may have side effects (Yes)	73.9%	66.9%	0.984	71.9% (69.2-74.5)	NA
Used only for acute illness (e.g. flu) (No)	45.3%	47.9%	0.443	47.2% (44.2-50.1)	NA
Overdose is possible (Yes)	53.3%	53.2%	0.982	53.3% (50.3-56.2)	NA

^a^2011 Hong Kong Census provisional figures; ^b^2006 Hong Kong Population By-Census; ^c^2004 Hong Kong Population Health Survey; NA = Not available.

#### ***COTC-related perceptions and preventive practices***

Respondents were also asked about their perceptions about the benefits of COTC use, the severity and susceptibility to COTC-related adverse events, and barriers to safe use of COTC. Respondents who had used COTC in the past year were also asked seven items about their COTC-related practices (see Table 2). Summative scores were calculated for each of these domains by summing the responses (Agree = 2, Not sure = 1, Disagree = 0) to each of the constituent items. For all summative scales, higher scores reflected greater levels of that domain (Table 3).

**Table 2:** COTC-related perceptions and preventive practices among users (n=789)

	**Response choices**		
	Agree	Not sure	Disagree
Perceived benefits			
At least as effective as western drugs	31.9%	22.8%	45.2%
Less side effects than western drug	69.1%	15.7%	15.2%
Cheaper than western drug	37.4%	18.8%	43.9%
Help reduce medical costs	42.1%	17.9%	39.9%
Safer than western drugs	39.4%	21.3%	39.3%
Helps people to stay healthy	54.9%	21.4%	23.6%
Perceived severity			
COTC poisoning is more serious than for western drugs*	34.2%	42.7%	22.9%
COTC poisoning due to drug interaction could be fatal	82.8%	12.0%	5.2%
COTC overdose could result in hospitalization	87.5%	7.7%	4.8%
Perceived susceptibility			
Easy to misuse COTC due to unclear instruction	33.8%	5.9%	60.4%
I am likely to experience COTC-related side effects	9.9%	8.4%	81.7%
I am likely to experience drug interactions with COTC	37.6%	24.8%	37.6%
Government has adequate regulation for COTC safety*	15.0%	20.6%	64.5%
Perceived barriers			
Making TCM doctor appointments for COTC advice is inconvenient	27.2%	31.7%	41.1%
TCM prescriptions are expensive	36.9%	25.5%	37.6%
Easy to find COTC information on the internet	16.2%	62.4%	21.4%
Physicians do not have reliable knowledge about COTC	71.8%	16.9%	11.3%
Pharmacists do not have reliable COTC knowledge	65.3%	17.8%	16.9%
COTC package instructions are inadequate or unclear	42.9%	18.1%	39.0%
Preventive practices**	Habitually	Occasionally	Never
Read COTC labels	66.8%	24.0%	9.3%
Read COTC package inserts	55.9%	32.0%	12.2%
Ask COTC information from retailers	13.2%	35.7%	51.1%
Search online for COTC information	3.9%	15.6%	80.5%
Ask Western MDs or pharmacists about COTC use	3.3%	12.6%	84.2%
Tell their medical doctor about TCM use	33.2%	20.6%	46.2%
Asked TCM practitioner about COTC	8.1%	22.0%	69.9%

*Item not included in summative scale score due to low item-total correlation.

**Original 5-point Likert responses were recoded as: Habitually (Always or Often), Occasionally (Sometimes or Seldom) and Never due to small cell counts.

**Table 3:** Comparison of COTC users (n = 789) who reported adverse events compared with those who did not report adverse events (AE)

	**Reported adverse events (n = 25)**	**No adverse events (n = 764)**	Unadjusted P–value*	**All COTC-users (n = 789)**
Conditions for past year COTC use	% (95% CI)	% (95% CI)		% (95% CI)
Cold/Flu	64.0% (43.8-84.2)	53.7% (50.1-57.2)	0.308	54.0% (50.5-57.5)
GI/Digestive problems	44.0% (23.1-64.9)	44.0% (40.8-47.9)	0.971	44.0% (40.3-47.2)
Musculoskeletal pains	76.0% (58.0-94.0)	42.7% (39.2-46.2)	0.001	43.9% (40.3-47.2)
“Qi” (氣) imbalances^a^	32.0% (12.3-51.7)	23.4% (20.4-26.4)	0.321	23.7% (20.7-26.7)
General health enhancement	20.0% (3.1-36.9)	13.5% (11.1-15.9)	0.351	13.7% (11.3-16.1)
Sleep problems	20.0% (3.1-36.9)	4.8% (3.3-6.4)	0.001	5.3% (3.8-6.9)
Skin and hair problems	8.0% (0.0-19.4)	4.1% (2.7-5.5)	0.333	4.3% (2.8-5.6)
Treating open wounds	8.0% (0.0-19.4)	3.8% (2.4-5.2)	0.287	4.1% (2.6-5.3)
Chronic respiratory problems	0.0% (0.0-0.0)	3.5% (2.2-4.8)	0.339	3.5% (2.2-4.7)
Slimming/Weight loss	8.0% (0.0-19.4)	0.9% (0.2-1.6)	0.001	1.1% (0.4-1.9)
Blood Pressure/heart conditions	4.0% (0.0-12.3)	0.4% (0.0-0.8)	0.012	0.5% (0.0-1.0)
Improving mental functioning/memory	8.0% (0.0-19.4)	0.0% (0.0-0.0)	<0.001	0.3% (0.0-0.6)
Sexual health/reproductive conditions	0.0% (0.0-0.0)	0.4% (0.0-0.8)	0.754	0.4% (0.0-0.8)
Vision problems	0.0% (0.0-0.0)	0.3% (0.0-0.6)	0.798	0.3% (0.0-0.6)
All other conditions^b^	4.0% (0.0-12.3)	1.7% (0.8-2.6)	0.392	1.8% (0.9-2.7)
Usual source(s) of COTC information				
TV	24.0% (6.0-42.0)	7.6% (5.7-9.5)	0.003	8.1%
Retailers	40.0% (19.4-60.6)	22.2% (19.0-24.0)	0.037	22.8%
Internet	4.0% (0.0-12.3)	5.7% (4.0-7.3)	0.724	5.6%
Newspapers	16.0% (0.6-31.4)	7.4% (5.5-9.2)	0.109	7.6%
Health professionals	8.0% (0.0-19.4)	11.6% (9.4-13.9)	0.570	11.6%
Friends and Family	52.0% (31.0-73.0)	42.9% (39.4-46.4)	0.377	43.4%
Magazines	20.0% (3.1-36.9)	4.9% (3.3-6.4)	0.001	5.3%
Drug labels/inserts	66.7% (39.4-90.0)	56.4% (51.2-58.8)	0.387	56.7%
Other sources (Books)	16.0% (6.0-31.4)	7.1% (5.2-8.9)	0.092	7.6%
	Mean (SD)	Mean (SD)		Mean (SD)
Practice score [out of-28]	12.2 (5.8)	9.12 (5.0)	0.002	9.22 (5.00)
COTC Knowledge scores [max = 6)	3.64 1.60)	3.52 (1.45)	0.712	3.52 (1.45)
Perceived benefits score [max = 12]	6.76 (3.40)	6.67 (3.01)	0.851	6.67 (2.99)
Perceived COTC AE severity [max = 4]	3.32 (1.14)	3.61 (0.87)	0.219	3.60 (0.88)
Perceived AE susceptibility [max = 6]	2.00 (1.99)	2.01 (1.71)	0.902	2.01 (1.72)
Perceived COTC info barriers [max = 12]	7.36 (2.31)	7.24 (2.20)	0.801	7.23 2.21)

*χ^2^ or t-test p-value.

^a^Qi (氣) imbalances refer to TCM concept of bodily vital energy disharmonies [38].

^b^Other conditions include: Headache, ear ache and multi-symptom conditions.

#### ***Proprietary Chinese medicine use patterns and reported adverse events among users***

Respondents who had used COTC in the past year were asked about the health condition for which the COTC product was used (see Table 3 for response categories). The list of conditions for which COTC products may have been used was compiled by licensed TCM practitioners and checked by research assistants against TCM products sold over-the-counter in local drug stores. In the pilot test, no other categories of COTC drugs were reported. COTC users were asked whether the product labels had clear instructions and whether they experienced any acute COTC-related adverse event within 2 days of use. Respondents were read a list of common adverse events that was initially compiled by two TCM practitioners with extensive clinical experience. In the pilot-testing of the instrument, respondents were asked about adverse events with extensive probing by the interviewers. Since no other adverse events were noted in the pilot study, it was concluded that all major categories were included in the survey instrument. The final survey instrument included an “other” response category for later respondents to report adverse events that may have been missed. Respondents who experienced adverse reactions were further asked about the dosage form of the COTC product, the condition for which the COTC product was used, and where they sought help for the adverse event (self-treatment, Western MD, TCM practitioner or other health professional). COTC users were also asked where they typically obtained information about COTC use (See Table 3).

### Statistical analysis

Descriptive statistics such as percentages and their respective 95% confidence intervals were reported for the prevalence data. Unadjusted analysis performed using chi-square, and t-tests were used to examine the associations between predictor variables the adverse event outcome variable. Summative scores were created for knowledge, TCM-related perceptions, and adverse event preventive behaviors domains by summing the responses in these domains. Items were removed from the scales to maximize the reliability coefficients (the final reliability coefficients ranged from 0.52 to 0.68). The scales were examined in the multivariable models after collapsing the scores into 3 levels: high score (score > interquartile range), mid-range score (within interquartile range) and low score (score < interquartile range).

Stepwise logistic regression was conducted to determine the factors associated with whether the respondent had experienced a past-year adverse event. For multivariable models, those socio-demographic variables which had p < 0.15 in the unadjusted analyses, and were first used as candidate variables for stepwise multivariable logistic regression models of socio-demographic factors only. A second multivariable logistic regression model (full model) was then conducted by also including attitudinal and behavioral variables as candidate variables that showed marginally statistically significant association with the outcome variable (p < 0.15) in unadjusted analyses (see Table 4). Model fit was examined using Hosmer-Lemeshow statistics. Models were checked for collinearity between variables by using variance inflation factor (VIF) statistics and by checking the standard errors of the covariates. All statistical analyses were conducted using SPSS for Windows version 16.0 (SPSS Inc, Chicago, 2007).

**Table 4:** Correlates of adverse events (AE) among over-the-counter Chinese medicine users (n = 789)

	**% reporting past-year adverse events**	**Unadjusted p-value**	Multivariable regression of socio-demographic factors*	Full multivariable regression model**
	**% (95% CI)**		**OR (95% CI)**	**OR (95% CI)**
Total sample of COTC users	3.2% (1.9-4.4)			--
Gender		0.146		
Male	2.2% (0.7-3.7)		1.00	--
Female	4.0% (2.1-5.9)		1.40 (0.56-3.50)	
Age		0.043		
18-44	1.9% (0.5-3.2)		1.00	--
45+	4.4% (2.4-6.4)		1.30 (0.49-3.49)	
Educational level		0.002		
F6 and higher	0.7% (0.0-1.6)		1.00	1.00
Up to F5 (grade 11)	4.8% (2.8-6.7)		7.43 (1.74-31.8)^2^	9.64 (2.20-42.3)^2^
Household income		0.291		
HKD 15,000 or more	4.3% (1.2-7.5)		--	--
HKD 0–14,999	2.7% (1.4-4.1)			
Health insurance		0.100		
Insured	4.3% (2.2-6.3)		1.00	--
Uninsured	2.2% (0.1-3.6)		1.34 (0.57-3.16)	
Employment		0.008		
Employed or FT student	1.8% (0.1-3.0)		1.00	--
All else	5.1% (2.7-7.5)		1.92 (0.79-4.64)	
COTC Knowledge levels		0.307		
High knowledge score (> IQR)	6.0% (0.1-12.0)			--
Score in interquartile range	2.9% (1.6-4.2)			
Low knowledge score (< IQR)	2.9% (0.0-6.9)			
Perceived benefits		0.141		
High benefits score (>IQR)	2.2% (0.3-4.1)			
Score in interquartile range	4.2% (2.3-6.0)			
Low benefits score (< IQR)	0.9% (0.0-2.8)			
Perceived barriers		0.472		
Low barriers score (< IQR)	3.2% (0.4-5.9)			--
Score in interquartile range	2.8% (1.3-4.2)			
High barriers score (>IQR)	4.9% (1.0-8.7)			
Perceived severity of COTC AE		0.260		--
High severity score (>IQR)	2.7% (1.4-4.0)			
Score in interquartile range	4.3% (0.9-7.7)			
Low severity score (< IQR)	7.7% (0.0-18.7)			
Perceived susceptibility to COTC AE		0.604		
High susceptibility Score (>IQR)	2.9% (0.4-5.4)			--
Score in interquartile range	2.6% (1.1-4.2)			
Low susceptibility score (< IQR)	4.1% (1.3-6.9)			
Preventive practices		0.008		
Low preventive practices Score (< IQR)	1.4% (0.0-3.3)			1.00
Score in interquartile range	2.5% (1.0-3.9)			2.59 (0.55-12.1)
High preventive practices score(>IQR )	6.0% (2.7-9.4)			6.47 (1.38-30.3)^1^
Exposed to any TCM warnings		0.650		
Yes,	2.9% (1.4-4.4)			--
No/Can’t recall	3.5% (1.3-5.6)			
Self-efficacy for obtaining reliable COTC info?		0.123		
Yes, have self-efficacy	2.4% (1.0-3.8)			1.00
No/Not sure	4.4% ( 2.1-6.7)			1.42 (0.59-3.42)
Usual source of COTC info				
Health professionals	2.2% (0.0-5.3)	0.570^§^		--
Internet	2.3% (0.0-6.9)	0.724^§^		--
Package labels & inserts	3.1% (1.2-4.6)	0.387^§^		--
Family & Friends	3.8% (1.8-5.9)	0.377^§^		--
Retailers	5.6% (2.2-9.0)	0.037^§^		1.98 (0.78-5.09)
Newspapers	6.7% (2.0-13.2)	0.109^§^		0.61 (0.11-3.26)
TV	9.4% (2.0-16.7)	0.003^§^		2.93 (1.01-8.50)^1^
Magazines	11.9% (1.7-22.1)	0.001^§^		3.32 (1.03-10.7)^1^
Books	6.9% (0.2-13.6)	< 0.001^§^		2.74 (0.84-8.90)

*Stepwise regression model using socio-demographic variables with p-value < 0.15 in the unadjusted analyses as candidate variables, non-significant covariates are shown with OR (95% CI) prior to removal from the final model.

**Stepwise regression model using educational attainment in addition to attitudinal and behavioral variables with p-value < 0.15 in the unadjusted analyses as candidate variables.

non-significant covariates are shown with OR (95% CI) prior to removal from the final model. Full Final model showed Hosmer-Lemeshow statistics = 0.595 and Variance Inflation Factor = 1.19.

§p-values for comparison with those not reporting those behaviors.

-- Variable not entered as candidate variable into multivariate model.

^1^p<0.05, ^2^p < 0.01.

## Results

The study sample was largely similar to the general Hong Kong population of adults, except that the study participants reported slightly higher levels of education and income (Table 1).

Of the study sample 2.3% (25/1100) reported a COTC-related adverse event in the past year.

The study sample showed moderate levels of COTC-related knowledge (Table 1). Although the majority of respondents were aware that COTC can have side effects and can interact with Western drugs, only about half were aware that COTC could interact with herbal TCM concoctions or that COTC overdosing is possible. There were no significant differences in knowledge levels between COTC users and non-users.

COTC-related perceptions and preventive practices among COTC users are shown on Table 2. Rather than cost-related aspects or beliefs about drug efficacy, the main stated benefits of COTC use were the fewer perceived side effects than western medications. While the vast majority of COTC users (>80%) agreed that COTC-related adverse events could cause hospitalization and even death, respondents demonstrated low perceptions of personal susceptibility to adverse events. The main barriers to greater COTC information seeking behaviors cited by respondents were the perception that reliable knowledge of COTC products could not be obtained from Western medical professionals and that package instructions on COTC products are unclear. COTC users reported a wide range of COTC-related health practices. While more than half would “habitually” read labels and package inserts, only a minority would routinely seek information about COTC from retailers or TCM practitioners, or tell their western medical doctors about their COTC use. Greater than 80% of respondents reported that they “never” sought information from Western health professionals or the Internet.

A comparison of COTC users who had experienced adverse events and those who had not is shown in Table 3. Among all COTC users, the most common health conditions which COTC was used for were: colds/influenza, gastro-intestinal problems, musculoskeletal pains, “Qi” imbalances (a TCM concept referring to bodily yin-yang disharmony) [42] and for enhancing general health. Use for other health concerns was infrequent. COTC users reporting adverse events were significantly more likely to have used COTC products for musculoskeletal pains, sleep problems, weight loss, cardiovascular conditions, and improving mental functioning. Respondents reporting adverse events were also more likely to obtain their COTC information from television, magazines and retailers. There were no statistically significant differences between the COTC users who experienced adverse events with those who did not with respect to perceived benefits, knowledge of COTC harms, perceived severity, perceived susceptibility, or perceived barriers to COTC information seeking (Table 3). When compared with their counterparts who did not report adverse events, however, respondents who reported adverse events had higher levels of preventive practices. Respondents who reported adverse events were also more likely to report mass media sources of COTC information. Nearly all of respondents citing “other” sources reported reading about TCM products in books, although those individuals were not health professionals.

**Figure 2:**
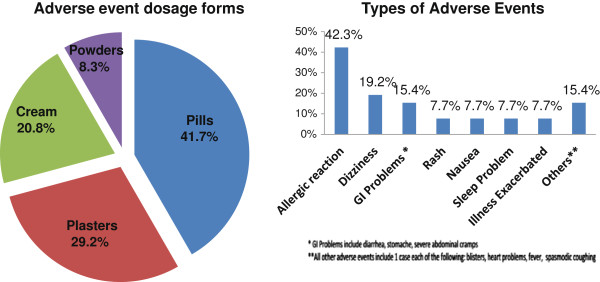
Description of adverse event dosage forms and types of adverse events.

Figure 2 demonstrates the description of the alleged adverse drug events reported. Among the 27 adverse events reported by 25 individuals in this study, the highest proportion of COTC reactions 37% were caused by pills/capsules (n = 10), while 25.9% were caused by plasters (n = 7), 18.5% by ointments/creams (n = 5), 11.1% from powder forms of COTC (n = 2), and none from syrups or tinctures. Allergic reactions, dizziness/disorientation, and gastro-intestinal symptoms (such as diarrhea, stomach ache and cramping) comprised nearly three-quarters of all the adverse reactions reported. Only about one-third of these individuals (n = 8) sought professional medical treatment. Of these eight respondents, allergic reactions accounted for 2 cases, while severe nausea, dizziness, sleep problems, stomach ache, fever, and exacerbation of influenza-like symptoms each accounted for one case (data untabulated).

The correlates of adverse events among COTC users are shown in Table 4. The unadjusted odds ratios for adverse events are also shown. Although factors such as older age were associated with adverse events, the multivariate analyses limited to socio-demographic factors showed that only those with less than grade 11 education were more likely (OR = 7.43) to have had an adverse event. The full multivariate analyses that also included knowledge, attitude, and practice variables as candidate variables, showed that those with less than grade 11 education (OR = 9.64), those who had greater adverse event preventive practice scores (OR = 6.3), and those who obtained COTC information from magazines (OR = 3.32) and television (OR = 2.93) were more likely to report COTC-related adverse events (p < 0.05). The correlates of adverse events in the entire study sample (COTC-users and non-users) were not substantively different to those reported for COTC-users only (data not tabulated). The proportion reporting adverse events was only 2.2% in the entire study sample (1.5% for males, and 2.9% for females). The multivariable logistic regression odds ratios were very similar to those shown in Table 4.

## Discussion

The prevalence of COTC-related adverse events (3.2% among COTC users) is slightly lower than the 4.6% reported among patients prescribed complementary and alternative medicine in a European hospital setting [34] and the 6.5% reported in a U.S. hospital-based study of conventional allopathic drugs [43] but considerably higher than the 0.4% reported in a 5.5 year prospective study on patients prescribed alternative medicine [32,44]. Methodological differences in study designs and study samples account for the wide range of adverse drug event rates. The results of this study, nonetheless, expose cases of COTC-related adverse events that had not been detected by previous clinic-based studies in Hong Kong. Since the majority of individuals with adverse events in our population-based study did not seek any type of professional medical treatment (and none sought emergency care), COTC-related adverse events reported primarily by emergency room clinicians appear to represent a small fraction of COTC-related adverse events in Hong Kong. Our results thereby indicate that COTC-related adverse events are an under-appreciated public health issue that may require greater scrutiny and improved surveillance. Since the list of adverse drug reactions includes many items which may be quite mild (such as minor skin irritation), the clinical significance of these adverse events is unknown and represents a limitation of the study. Nonetheless, approximately 1% of the COTC users (n = 8) had an adverse reaction for which they sought medical treatment, giving a rough estimate of more severe adverse events in the population. Although a previous study conducted on emergency room patients in Hong Kong reported that rashes and systemic allergic reactions comprised over 90% of the adverse events [35,45], our study found that these conditions only represented about half of the adverse events in the general population while dizziness, nausea, vomiting, and gastro-intestinal problems comprised most of the remaining cases. Adverse events such as sleep disturbances, exacerbation of existing illnesses, and heart-related problems that were previously unreported in the COTC literature were also shown in this study.

Despite government efforts to promote safe COTC use through more stringent labeling requirements, over one third of respondents still find COTC labels to be unclear. Part of the reason for the ambiguity is that many of the listed drug actions, such as “dispelling dampness” or “normalizing the gall-bladder”, require advanced understanding of TCM concepts. In our study, the observed preference for unreliable sources of COTC information (e.g. mass media magazines) was shown to be the primary behavioral risk factor for COTC-related adverse events. Health information presented in the mass media is often inaccurate or incomplete [43-47]. Even among Hong Kong Chinese, a population with a long tradition of TCM use, TCM-related misconceptions and low risk perception of COTC harms are pervasive. Hence, strategies promoting safe COTC use must also include raising public awareness of alternative medicine safety. Our study corroborated findings from other regions that showed low risk perceptions related to complementary and alternative medicine use [25,48].

The importance of increasing risk perception is particularly necessary since the inclination to self-manage health problems is strongly associated with alternative medicine use [49,50]. In our study, COTC-users self-medicated without consulting TCM practitioners. Case reports about the adverse effects from misuse of complementary and alternative medicine from self-treatment abound in the medical literature [51-53]. It is particularly noteworthy that a large proportion of adverse event victims reported books as a common source of COTC information, even though none of those individuals were health professionals. Self-management of health using COTC among people untrained in TCM precepts has great potential for inappropriate COTC use, particularly since TCM users in Hong Kong possess lower educational levels [54]. TCM treatments rely upon holistic diagnosis of the underlying syndrome while the prescribed treatments for a particular symptom may vary greatly between individuals.

In addition to addressing pervasive COTC misconceptions, there appears to be a need to reduce barriers to obtaining reliable drug safety information. Past surveys of Hong Kong pharmacists [55] and western-trained medical doctors [56] demonstrated a low level of TCM knowledge. Greater dialogue between TCM manufacturers, retailers, and Western health professions is required to develop effective safety measures for COTC users.

The trend towards greater alternative medicine use worldwide necessitates not only stringent labeling regulations and better consumer risk communication, but also improved surveillance of adverse events. The much higher rates of adverse events uncovered by this population-based study mirrors findings from other countries which found self-reported adverse events exceed those noted by doctors [57-59]. These findings indicate that adverse event reporting in an outpatient setting (e.g. web-based reporting or adverse event hotlines) should be explored. Improved surveillance of complementary medicines should be prioritized by governments in order to provide more comprehensive safety information for health professionals and consumers.

The main limitation of this study is the lack of clinical validation of self-reported adverse events some of which may have been unrelated to COTC use. Alternatively, it is also possible that some COTC-related adverse drug reactions may have not been recognized as such by users. Even among the valid cases of COTC adverse events that were captured by the study, it was not possible to determine whether poor drug quality, product misuse, or drug interaction was the underlying cause of the adverse event. Moreover, the direction of the positive association between adverse events and greater information seeking behaviors (e.g. reading labels) is unclear. However, recall biases are likely to be moderate due to short time frame of the recall period (past year). Lastly, the reliability of the summative scales (Cronbach’s α ranging from 0.52 to 0.68), indicated that these scales should only be used for exploratory purposes.

## Conclusions

Despite the limitations noted above, our study can inform drug policy for other countries that are implementing complementary medicine regulation. Although the US and European Union have enacted food and drug regulations for complementary and alternative medicine products that will reduce adverse events from poor drug quality, the lack of understanding of the potential harms of COTC self-medication and the inaccessibility of reliable information still pose major obstacles to drug safety.

### Ethics

Appropriate IRB was solicited from the Chinese University of Hong Kong Ethics Committee and participants’ privacy was honored. Furthermore, informed consent was elicited from each patient after thorough explanation of the risks and benefits of participating in this project. Our protocol abided by the Declaration of Helsinki.

## Competing interests

There is not any financial or non-financial competing interest in this research. We have not received any reimbursement, fee, funding, or salary from any organization that many in any way gain or lose financially from the publication of this manuscript, either now or in the future.

## Authors’ contributions

JHK designed the project and participated in data collection, analysis, interpretation of data, and writing of the manuscript. VCHC consulted the team and participated in data collection, analysis, interpretation of data, and writing of the manuscript. JCOL participated in data collection, analysis, and interpretation of data. TW helped with instrument refinement and drafting of the manuscript. EMSK helped in writing the manuscript and in data collection. All authors read and approved the final manuscript.

## Pre-publication history

The pre-publication history for this paper can be accessed here:

http://www.biomedcentral.com/1472-6882/13/336/prepub
